# The underlying factors of excess mortality in 2020: a cross-country analysis of pre-pandemic healthcare conditions and strategies to cope with Covid-19

**DOI:** 10.1186/s12913-021-07169-7

**Published:** 2021-11-05

**Authors:** Nikolaos Kapitsinis

**Affiliations:** Cardiff University, Business School, Aberconway Building, Colum Dr, Cardiff, CF10 3EU UK

**Keywords:** Excess mortality, Covid-19, Healthcare expenditure, Primary healthcare, Test and trace, Lockdown, I11, I18, I3

## Abstract

**Background:**

Government responses to the pandemic varied in terms of timing, duration, and stringency, seeking to protect healthcare systems, whose pre-pandemic state varied significantly. Therefore, the severity of Covid-19 and, thus, excess mortality have been unequal across counties. This paper explores the geography of excess mortality and its underlying factors in 2020, highlighting the effects of health policies pre-pandemic and strategies devised by governments to cope with Covid-19.

**Methods:**

Excess mortality is estimated for 79 high, medium and low-income countries. The factors of excess mortality are examined employing median quantile regression analysis.

**Results:**

Health privatization, healthcare underfunding, and late implementation of containment and mitigation strategies were powerful drivers of excess mortality. By contrast, the results suggest a negative association of excess mortality with health expenditure, number of doctors and hospital beds, share of population covered by health insurance and test and trace capacity.

**Conclusions:**

The evidence highlights the importance of sufficiently funded healthcare systems with universal access and strong primary healthcare in the battle against the pandemic. An early response to Covid-19, including borders’ controls and a strong test and trace capacity, could improve epidemiological surveillance and minimize excess mortality, with stringent and lengthy lockdowns not providing a significant benefit.

## Introduction

Covid-19 has wrought havoc in most countries since early 2020. In addition to the health crisis implicating millions of deaths, whole economies were wound down and entire countries have been grinding to a halt with long periods of general confinement (‘lockdown’). However, the rate of Covid-19 deaths present a high degree of diversity across countries [23, 38]. Some countries have witnessed very high mortality rate, including Peru with 578 Covid-19 registered deaths per 100 k people by 6th July 2021, Hungary with 311, Brazil with 245, UK with 187, and USA with 186 [19]. By contrast, other countries have reported a very low number of deaths, such as Vietnam with 0.09 deaths per 100 k inhabitants, New Zealand with 0.5, Iceland with 8.7, and Taiwan with 3 deaths per 100 k people. Apart from the different methods adopted by each jurisdiction to register Covid-19 deaths, resulting in many fatalities being under-reported, the outbreak has stretched the national healthcare systems to their limits, undermining their capacity to offer vital health services, possibly adding to the rise in deaths and entailing high excess mortality [17]. Many societies are still, 18 months after the outbreak of the pandemic, facing severe restrictions, with disruption of social activities, while in other countries life has almost returned to normal, with minor social curbs [47]. It is striking that the latter are likely to have recorded low numbers of Covid-19 deaths [50, 55]. All the above underpin the significant diversification of the Covid-19 impact across countries and highlight the importance of strategies devised by the national governments to handle the pandemic.

This paper examines the geography of excess mortality and its underlying factors in 2020, focusing on the effects of the long-term health policies pre-pandemic as well as the urgent policy measures put forward by governments to cope with Covid-19, which present a considerable diversity in terms of timing, duration and stringency. By scrutinizing the relationship of the state of healthcare and the strategies adopted to handle the pandemic with excess mortality, this paper seeks to evaluate the preparedness and health responses of different countries. It also aspires to explain why countries implementing less severe social curbs have witnessed a limited Covid-19 impact and highlight what has been done differently. This paper delves into the role of national policies, since regardless of the social, economic and cultural framework of each country that has proved to influence the severity of the pandemic [20, 38], these strategies as devised by the state could act as a game-changer.

The paper at hand seeks to make three important contributions. First, it is noted that the emerging literature on the pandemic impact and the effectiveness of government responses has focused mainly on Covid-19 infection, mortality or fatality rate [18, 20, 34, 38], overlooking the potential importance of excess mortality and thus failing to comprehensively grasp the wider impact of the pandemic. The main novelty of this paper pertains to stressing the importance of excess mortality. This approach offers an integrated analysis of the broader effects of Covid-19 facilitating a comprehensive understanding of the unrelenting pressure faced by the national healthcare systems during 2020, the first year of the pandemic. Excess mortality is argued to capture the Covid-19 deaths officially reported, the fatalities of the virus that have not been registered, but also the deaths occurred due to other diseases that might have been prevented if the pandemic had not overwhelmed healthcare systems [12].

The research agenda on factors underlying the Covid-19 impact has exposed travel restrictions [26], quarantine measures [30], and healthcare system resources [18]. This paper aspires to expand knowledge on the inquiry of the Covid-19 effects by testing the significance of these factors, but also examining the role of determinants that have not been studied yet, including health expenditure, access to healthcare and health privatization trends, while adding control variables of the socio-economic framework, such as the poverty rate and social trust. Chiefly, it evaluates the importance of the timing of the government strategies’ implementation. Therefore, the paper comprehensively evaluates the preparedness of healthcare systems and efficiency of policy responses, while also accounting for important socio-economic features. It sheds light on the broader impact of all these factors based on their interaction, employing multiple regression analysis.

Third, most scholars evaluating the efficacy of policy responses to Covid-19 have focused on single case study countries [13, 26, 30, 55]. This paper moves beyond these research efforts, focusing on a large group of countries, including high, medium and low-income economies, thus providing a comparative analysis of preparedness of healthcare systems and policy efficiency. This is of great importance, when considering the substantial inequality in healthcare conditions between high- and low-income countries, but also the significant disparities among social classes in access to healthcare services, particularly in low-income countries, according to Word Health Organization (WHO) [52].

In what follows, Background: healthcare conditions and strategies to cope with the pandemic section develops the conceptual framework intertwining pandemics with space and the policies pursued to cope with Covid-19, before moving on to the methodology (Methods section). Results section analyses the factors affecting excess mortality in the study countries, while Conclusion section concludes. Appendix presents additional information about the variables.

## Background: healthcare conditions and strategies to cope with the pandemic

The emergence of pandemics has significantly increased during the last decades [27]. Social, economic and behavioral restructurings, including the escalation in climate change impacts, growth in international mobility and trade, and the application of rolling-back welfare policies undermining the operation of healthcare systems, have paved the way for the development of new pandemics [32]. Ebola and SARS are representative examples of recent infectious diseases. Space is considered significant for the analysis of pandemics. A contagious disease can be transmitted locally by spatial diffusion, radiating out of an epicenter [27]. Viruses could expand over a longer distance, leading to international spread, via trade and air travel. The transmission of the virus is a complicated socio-spatial process driven by spatial and social proximity, social interactions and concentration of people [6]. The latter are affected by aspects of the social, economic and political framework. The geographically uneven increase in mobility and interconnectedness in the new globalized society could assist in accelerating the development of a pandemic [24]. Socio-spatial and demographic inequalities should be also considered. Pandemic effects are not equal among individuals of different age, with elderly people being particularly susceptible to the disease [10]. Alongside the uneven impact in terms of age, the impact of a pandemic is unequal across social classes, since the poorest appear to be more vulnerable [4]. This paper delves into the strategies devised by governments to contain or mitigate the pandemic, presenting a noticeable geographical variation [32]. It also scrutinizes the state of healthcare, whose conditions are also diversified across space [40].

Healthcare systems that have historically been well funded and supported could assist in tackling a pandemic more efficiently [33]. There are four main healthcare models in terms of funding and access, as summarized by Wallace [51]. First, the Beveridge model that is government-financed through tax contributions providing for all citizens. Second, the Bismarck model is based on an insurance system, which is often co-financed by workers and employers, with healthcare providers frequently being private. Third, the National Health Insurance model comprises private hospitals and doctors, while being financed by a government insurance scheme funded by all taxpayers. Finally, the ‘out-of-pocket’ model, which is the most common, met in the medium and low-income countries, with healthcare services offered only to people affording to pay.

The rolling-back of welfare states during the neoliberal era is considered to be crucial for the battle against a pandemic. The trend observed since the 1980s indicates that state responsibility towards healthcare has moved sideways, with national governments pursuing a ‘hollowing-out the state’ strategy [45]. States have receded from one of their major roles as providers of universal health coverage, choosing not to sufficiently fund the healthcare systems accelerating privatization, with health services increasingly being outsourced to private firms or public-private partnerships [40]. Such policies have undermined public healthcare systems, exposing them to the risk of being overwhelmed under special circumstances and amplifying pre-existing socio-spatial inequalities in access to public health, particularly in light of the substantial cuts imposed in health expenditure in managing the 2007/08 global economic crisis [21].

Governments have responded to Covid-19 by implementing non-pharmaceutical and behavioral interventions, introducing various restrictions on social and economic life to contain or mitigate the pandemic [26]. The main containment policies are associated with effective and up-to-date epidemiological surveillance, including test, trace and isolate (TTI) the infected population and testing air passengers upon arrivals [50]. Mitigation measures involved socio-economic life curbs, such as closure of firms, remote working, border restrictions, curfews, and state-at-home orders or lockdowns [47]. According to recent evidence, containment strategies proved to be more efficient in drastically controlling the spread of the virus, since authorities adopting such policy measures have witnessed less infections than countries implementing mitigation policies [31]. Mitigation interventions are likely to achieve the optimal outcomes when accompanied with measures that are applicable to the entire population, such as physical distancing between people [34].

## Methods

To examine excess mortality factors, a cross-sectional multiple regression model is employed, estimating the association of different policies, pre- and post-pandemic, and specific socio-economic features with excess mortality. The latter is defined as ‘the mortality attributable to the crisis, above and beyond deaths that would have occurred in normal conditions’ ([8]: 5). The model takes the following form:

$$ {Y}_c={a}_0+\sum \limits_{\lambda =1}^n\left({\alpha}_{\lambda }{X}_{\lambda, c}\right)+{\varepsilon}_c $$

where *Y*_*c*_ is the dependent variable excess mortality in 2020 in country *c* under consideration, *X*_*λ,c*_ is the set of *λ* independent-explanatory variables for country *c*, *a*_*λ*_ is the set of the coefficients of the *λ* independent variables, *a*_*0*_ is the value of excess mortality when the independent variable is zero and *ε*_*c*_ is the error term that considers unobserved factors.

Excess mortality in 2020 is employed as the dependent variable and is preferred to infection and mortality rate. Covid-19 infection rate (positive tests per capita) could be prone to bias since countries adopt dissimilar testing policies [38]. Some authorities put forward an aggressive testing strategy, while other countries tested just the serious cases [44]. Several issues limit the capacity of Covid-19 mortality rate to capture the impact of the pandemic. First, there are different criteria for death registrations, as some authorities officially report Covid-19 deaths after a test has been made, whereas others publish data for deaths of persons suspected to have been infected by the virus. Second, figures of Covid-19 deaths occurred in some countries are sourced only from hospitals, while other countries also incorporate deaths outside healthcare units [20]. Finally, timing appears to be an issue since some jurisdictions record severe delays to publish Covid-19 deaths.

Considering that testing strategies and death registrations vary significantly, excess mortality appears to ease comparisons among countries. Scrutinizing excess mortality creates an opportunity to have a better understanding of the broader implications of the pandemic since it captures Covid-19 deaths officially reported and deaths from the virus that have not been registered. It also takes into account indirect deaths from other diseases that might have been prevented if the healthcare systems had not been overwhelmed due to the pandemic. This is a major issue, particularly for healthcare systems of limited capacity and preparedness to cope with a pandemic, since thousand deaths occurred in 2020 from diseases aside from Covid-19 could have been averted in a ‘normal’ year [12].

Excess mortality in 2020 as % compared to the average mortality from 2015 to 2019 is the dependent variable. The figures about annual deaths for the years between 2015 and 2020 in 79 countries are collected from the *World Mortality Dataset* [22] and from national health authorities.^1^ To construct the index of excess mortality or p-score, the following formula is estimated:

$$ {p}_c=\frac{m_c^{2020}-{m}_c^{2015-2019}}{m_c^{2015-2019}}\ast 100 $$

where *p*_*c*_ denotes the p-score (excess mortality) in country *c* under consideration, ${m}_c^{2020}$ is the number of deaths in 2020 in country *c*, and ${m}_c^{2015-2019}$ is the average number of deaths between 2015 and 2019 in country *c.* Considering the geographically unequal distribution of Covid-19 fatalities [2, 23], the distribution of the dependent variable proved to be not normal, after testing with the Shapiro-Wilk test. To address this issue, alongside heteroskedasticity and outliers, the author ran a median quantile regression model. The regression model is developed to examine the statistical relationship between excess mortality and the following explanatory variables.

Better funded healthcare systems provide higher quality of services and have greater capacity to cope with the pandemic, without being overwhelmed [33]. Health expenditure as % of GDP is captured (Table 1) and is likely to be negatively correlated with excess mortality. To capture the substantial cuts in health expenditures to cope with recession that national states have imposed in the aftermath of the 2007/08 crisis [40], per capita health expenditure % change between 2008 and 2018 is employed and is expected to be positively related to excess mortality. Finally, the variable of primary healthcare expenditure per capita is expected to be adversely correlated to excess mortality [12]. Adequately funded healthcare systems exhibit adequate level of healthcare resources. The numbers of both public and private hospital beds, as well as doctors and nurses are expected to be negatively associated with excess mortality.

**Table 1:** Variables and their definitions

	Variable name	Construction and source
**Healthcare funding**	HEAGDP_(log)_	Health expenditure as % of GDP in 2018 (sourced from World Bank)
	PRIMARPC_(log)_	Primary healthcare expenditure per capita in current $ in 2018 (sourced from WHO)
	CHAHEAPC	Per capita health expenditure % change between 2008 and 2018 is employed (sourced from World Bank)
**Healthcare resources**	DOC_(log)_	Doctors per 100 k people in 2018 (collected from World Bank)
	BEDS_(log)_	Public and private hospital beds per 100 k people in 2018 (collected from World Bank)
	NURS_(log)_	Nurses and midwives per 100 k people in 2018 (collected from World Bank)
**Healthcare access**	HEALTHINS_(log)_	Share of population covered by health insurance (sourced from Our-World-in-Data in [16])
	PRIHEAPC_(log)_	Domestic private health expenditure per capita in purchasing power parity (PPP), current international $ (sourced from WHO)
	PRIHEA_(log)_	Domestic private health expenditure as % of current health expenditure (sourced from WHO)
	BEVER	Dummy variable, where 1 = Beveridge model, 0 = no Beveridge model
**Covid-19 mortality**	COVDEATH_(log)_	Covid-19 deaths per 100 k people in 2020 (collected from Our-World-in-Data)
**Covid-19 testing**	INDTTI_(log)_	Average index of TTI (INDTTI) for 2020 (ranging from 0 to 2), calculated using data from the Oxford COVID-19 Government Response Tacker (OxCGRT) [15]. OxCGRT published data for the index for each day in 2020. The average index refers to the mean for 2020. Higher values of the index correspond to strong, comprehensive, effective systems of TTI, with contact tracing for all identified cases.
	COVTESTS_(log)_	Covid-19 tests undertaken during 2020 per 100 k people (sourced from Our-World-in-Data)
**Containment and mitigation strategies**	DAYSWORE _(log)_	Days of any measure about workplaces (recommending or requiring closing or remote working) during 2020 (sourced from OxCGRT)
	DAYSLOCK _(log)_	Days of lockdown in 2020, with a requirement of not leaving house apart from a few exceptions (sourced from OxCGRT)
	INDLOCK_(log)_	Average index of stay-at-home order in 2020, ranging from 0 to 3. Figures are derived from OxCGRT, which published figures for the index for each day in 2020. The average lockdown index refers to the mean for 2020. High values of the index pertain to highly stringent lockdown
	CASBORCLO_(log)_	Covid-19 cases per 100 k people when a ban on all regions or total border closure was implemented (sourced from OxCGRT)
	DAYSTEDEA	Number of days between the detection of first Covid-19 case and first death (sourced from Our-World-in-Data)
	DAYDEARES	Number of days between the implementation of any policy measure and first death (sourced from Our-World-in-Data)
**Control variables**	AGE_(log)_	Median age in 2020 (figures are retrieved from the United Nations Database)
	GDPPC_(log)_	GDP per capita, in PPP, current international $, in 2019 (retrieved from the World Bank)
	POV_(log)_	Poverty headcount ratio at national poverty lines in 2019 (% of population) (World Bank data) [56]
	RULELAW	Rule of law index in 2019 (World Bank data) [56]
	PERFAMREL_(log)_	Personal and family relationships index in 2020 (sourced from Legatum Institute) [29]
	SOCTRUST_(log)_	Trust among citizens index in 2020 (sourced from Legatum Institute) [29]
	REL_(log)_	Level of weekly worship attendance in 2017 (derived from Pew Research Centre) [41]
	INS	Dummy variable, where 1 = insular, 0 = non-insular
	POPDENS_(log)_	Population density calculated as people per km^2^ in 2019 (United Nations data)
	EXPPC_(log)_	Value of exports per capita variable in 2019 (World Bank data) [56]

Domestic private health expenditure variables are also employed to capture the class barriers against access to healthcare services posed by the increase in health services’ provision by private bodies [45]. In relative terms, countries adopting the Beveridge model exhibit higher possibility for universal health coverage, with all the citizens having access to government-funded healthcare, regardless of income or employment status [51]. These countries are expected to record lower excess mortality. The share of population covered by health insurance is expected to be adversely associated with excess mortality.

Considering the severe effects of the pandemic [17], Covid-19 deaths are likely to be positively correlated with excess mortality. Testing is among the main tools to handle the pandemic [34, 35]. The variables of Covid-19 tests undertaken per 100 k people and average index of TTI for 2020 are expected to be negatively associated with excess mortality.

Different non-pharmaceutical interventions are tested regarding timing, duration and stringency. The variable of Covid-19 cases per 100 k people when a ban on all regions or total border closure was implemented is expected to be positively related to excess mortality since it indicates a late response against the pandemic. The timing of epidemiological surveillance is explored by employing the variable of number of days between the detection of first Covid-19 case and first death. Workplace closures is tested in terms of duration, namely the total days of any measure about workplaces (recommending or requiring closing or remote working) during 2020.

Among the strategies devised to handle the pandemic, ‘lockdown’ or ‘general confinement’ has been the most widespread term, although there is not a commonly accepted definition, since various actors have been using it referring to different social curbs. Following Plümper and Neumayer [42], compulsory stay-at-home order (or general confinement) is considered the strategy that is most closely related to the term of lockdown. To test the duration of lockdown in each country, the author estimated the total days of lockdown in 2020. Lockdown stringency is tested by calculating the average index of stay-at-home order in 2020. Finally, in order to assess the wider impact of timing, the variable of number of days between the implementation of any policy measure and first death is used.

Control variables are also employed to test the impact of particular socio-economic factors on excess mortality. Demographics are controlled by estimating the median age. Population density is expected to be positively correlated to excess mortality. Global interconnectedness is tested by employing the value of exports per capita. Economic dynamism is controlled by using the variable GDP per capita. Poverty level is examined by using poverty rate. Physical geography might be crucial, with insular countries possibly having an advantage since it was easier to control their borders [47]. Factors of the formal and informal institutional arena were tested by employing the variable of rule of law, trust among citizens, personal and family relationships and religious trends.

Seeking to explain the aggregate impact of these factors on excess mortality, the model has nine different versions, with each simulation including different explanatory variables. The author builds on the main assumption that healthcare system resources, healthcare funding (primary and overall), access to healthcare, Covid-19 fatalities, Covid-19 testing, and strategies against the pandemic, are the six key factors in explaining the geographically uneven excess mortality. Therefore, explanatory variables associated with these factors are included in every simulation, apart from the last one that aspires to control the impact of variables excluding Covid-19 mortality and access to healthcare. The author devises alternative estimation strategies to test the effects of a wide set of these key parameters and to assess their stability and robustness across various estimation methods. Thus, different variables are used for specific factors, such as Covid-19 tests per 100 k people and average index of TTI to control the impact of testing policies. In this way, the bias of the results is minimized and their validity increases. Control variables are progressively added to each version of the model, thus increasing the results’ robustness.

To correct heterogeneity and normalize the variables, the log is taken for all the parameters, apart from them which are dummy or whose observations have negative values. The correlation matrix did not show high correlation among the parameter estimates included in the same simulation (Table 3 in Appendix), highlighting no biased estimates caused by multicollinearity. These controls increase the validity of the model and confirm that it is not threatened from a highly skewed distribution of the variables. Table 4 in Appendix presents the descriptive statistics of excess mortality and the parameter estimates.

This regression analysis focusing on excess mortality constitutes a significant advancement, in comparison to recent accounts that tend to explain the impact of the pandemic based on Covid-19 mortality, fatality or infection rate. Regarding limitations, while some of the above factors are likely to affect mainly the Covid-19 infection rate, the selected index of excess mortality is argued to effectively capture such impacts, considering that high Covid-19 transmission is associated with high death rate and thus higher excess mortality [17]. Another limitation refers to data availability about deaths, since excess mortality for some countries was not estimated as the deaths in 2020 compared to the average of 2015-2019 but compared to the average of specific years within this period that figures were available. Additionally, some countries had recorded increasing mortality trends before 2020, particularly in poor countries in Africa and Asia or ageing societies in Europe. Therefore, the excess mortality variable could be prone to bias in these societies. Notwithstanding the above, the paper is argued to capture the effects of different factors on mortality in these countries, even though some of them have not been strongly affected by the pandemic, as it employs the variable of mortality in 2020 in excess of the average in the previous 5 years. On balance, the author strongly believes that the negative effects of these limitations are harnessed through the multiple robustness controls that improve the validity of the results, enhance the rigor of the research arguments and provide valuable holistic insights into the broader impact of Covid-19 across countries with different level of economic growth.

## Results

### Excess mortality trends

**Fig. 1:**
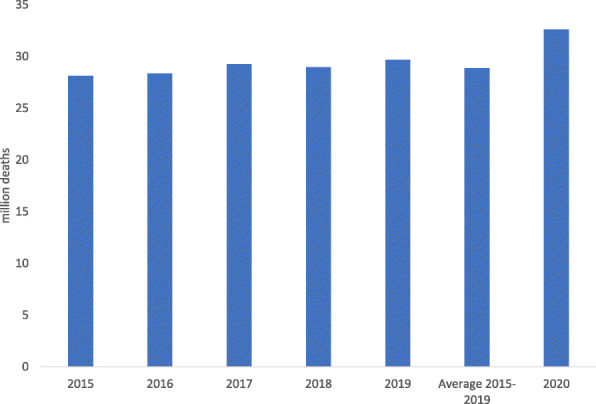
Total number of deaths in the study countries. Source: World Mortality Dataset and own elaboration

Figure 1 reveals the total number of deaths in the 79 study countries from 2015 to 2020. Around 3.7 million more deaths were reported under these jurisdictions in 2020 compared to the average fatalities between 2015 and 2019, witnessing a 13% excess mortality. This amount is estimated to be much higher at a global level when figures from all the countries are added, an attempt that was not possible in July 2021 since several countries had not published death figures for 2020.

**Fig. 2:**
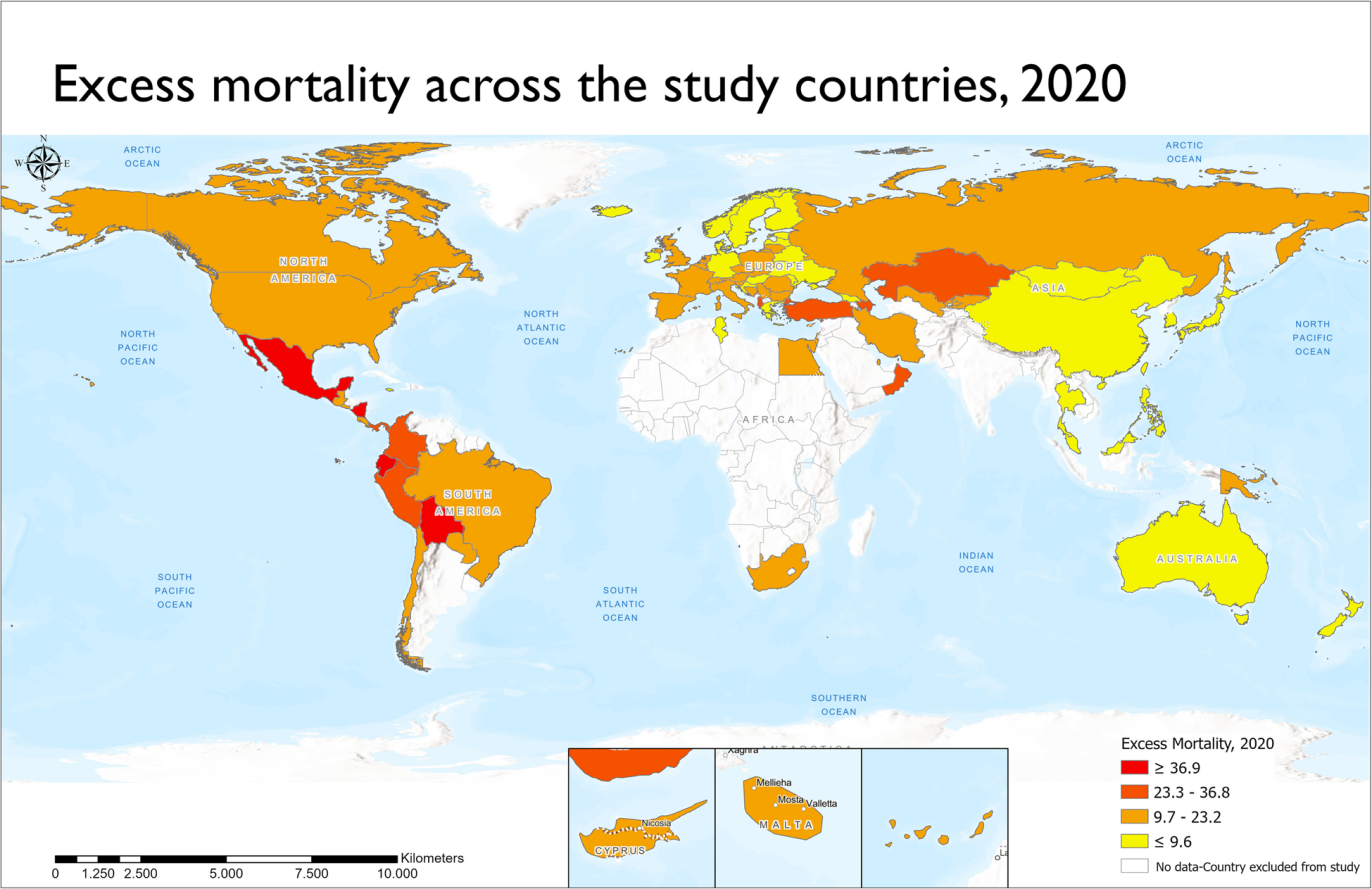
Excess mortality across the study countries, 2020. Source: World Mortality Dataset and own elaboration

The map in Fig. 2 indicates a significant increase in excess mortality during 2020 for the vast majority of the study countries. Deaths in 2020 were higher than the average mortality between 2015 and 2019 in 77 out of the 79 study countries (Table 5 in Appendix), while excess mortality (p-score) was lower than 2% only in 8 countries. The map also highlights an important variation of excess mortality across the countries examined. The five countries with the highest excess mortality include Mexico (50.4%), Nicaragua (50.1%), Ecuador (49.3%), Bolivia (48.8%) and Azerbaijan (32.6%), with four of them being in Central and South America. By contrast, the bottom-five countries in terms of excess mortality are spread across several continents: Mongolia (− 4%), Australia (− 0.1%), Jamaica (0.1%), Norway (1%) and New Zealand (1.1%).

**Fig. 3:**
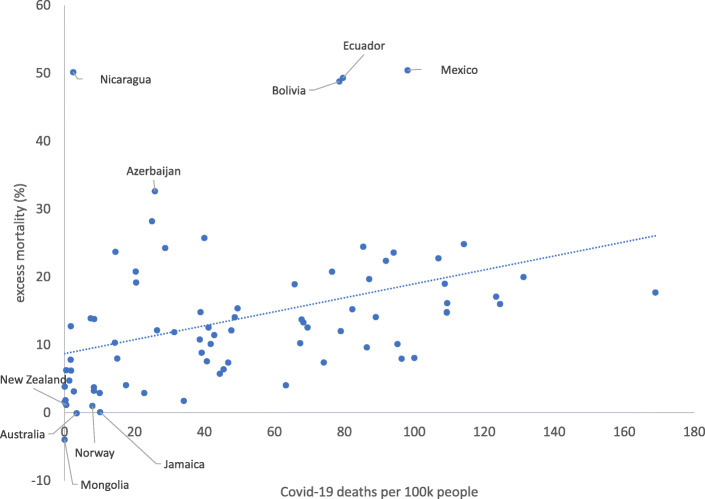
Correlation between excess mortality and Covid-19 deaths per 100 k people across the study countries, 2020. Source: World Mortality Dataset and own elaboration

Covid-19 constitutes the main cause for this significant rise of deaths in both economically developed and underdeveloped countries (Table 5 in Appendix). Figure 3 demonstrates the positive correlation between Covid-19 deaths per 100 k people and excess mortality in 2020. Among the study countries, the ones with the highest Covid-19 mortality rate are in Europe (Belgium with 169, Slovenia with 131, Bosnia and Italy with 123 Covid-19 deaths per 100 k people) and one in South America (Peru with 114), based on figures from Our-World-in-Data. By contrast, the countries with the lowest Covid-19 mortality are in Asia, where the pandemic started (Taiwan and Mongolia with 0.03, Thailand with 0.1, China with 0.3 and Singapore with 0.5 Covid-19 deaths per 100 k people).

None of the countries in the top-five regarding Covid-19 deaths per 100 k people is among the top-five countries in terms of excess mortality, highlighting the significance of under-reporting of Covid-19 deaths [13, 28]. For instance, a comparison between the excess deaths in Egypt in 2020 to the average deaths from 2015 to 2019 (72,620) indicated a tenfold difference to the officially reported Covid-19 fatalities in 2020 (7630). Almost 126,000 Covid-19 fatalities were reported from Mexican health authorities in 2020, equal to just 35% of the excess deaths in 2020 (352,000 more deaths were recorded in 2020 than the average fatalities between 2015 and 2019). The Covid-19 deaths reported in 2020 in Poland (28,550) accounted only for the 34% of the excess fatalities in this year (83,530). Finally, the USA registered 351,000 Covid-19 fatalities in 2020, but 625,000 excess deaths. Overall, while the 79 countries reported 3.7 million fatalities in 2020 in excess of the average 2015-2019, only 1.5 million were reported as Covid-19 deaths in 2020.

The testing system in many countries has been poor, resulting in thousands of deaths due to Covid-19 not being registered in official databases, with this explaining part of the significant discrepancy between Covid-19 deaths and excess mortality figures [28]. Apart from the direct or indirect impact of the pandemic, excess mortality trends during 2020 increase in significance when considering that the annual number of deaths from other diseases might have seen declining trends due to the increased health protection measures and social curbs. The latter have resulted in a significant fall of deaths from influenza in 2020, resulting from the physical distancing measures implemented [53].

Table 2 reveals the nine hierarchical models and the results of the regression analysis of the factors underlying excess mortality in 2020. The first value indicates the coefficient of the predictor. The value in the parenthesis shows the probability value that determines the statistical significance of the explanatory variable at each level (1, 5%, or 10%).

**Table 2:** Results of median quantile regressions

	Variables	(1)	(2)	(3)	(4)	(5)	(6)	(7)	(8)	(9)
**Healthcare funding**	HEAGDP_(log)_	− 0.738*** (0.004)		− 0.728** (0.011)	−.807*** (0.000)		− 0.830*** (0.003)		− 0.595** (0.024)	
	PRIMARPC_(log)_		−0.264* (0.092)							
	CHAHEAPC					0.001 (0.406)				− 0.002*** (0.000)
**Healthcare resources**	DOC_(log)_		−0.139 (0.580)	−0.234 (0.244)	− 0.333** (0.047)	0.189 (0.366)		−0.045 (0.821)		
	BEDS_(log)_						−0.223* (0.094)			0.148 (0.230)
	NURS_(log)_	−0.136 (0.181)							−0.035 (0.758)	
**Healthcare access**	HEALTHINS_(log)_	−0.192 (0.176)		−0.177 (0.261)		−0.412** (0.009)			−0.213 (0.130)	
	PRIHEAPC_(log)_		0.345 (0.191)							
	PRIHEA_(log)_				0.473** (0.006)					
	BEVER						−0.250*** (0.003)	−0.023 (0.790)		
**Covid-19 mortality**	COVDEATH_(log)_	0.387*** (0.000)	0.395*** (0.000)	0.365*** (0.000)	0.436*** (0.000)	0.330*** (0.000)	0.298*** (0.000)	0.349*** (0.000)	0.3167** (0.000)	
**Covid-19 testing**	INDTTI_(log)_	−0.318* (0.054)		− 0.490*** (0.004)	−0.605*** (0.000)	− 0.412** (0.009)		− 0.409** (0.007)		− 0.388*** (0.003)
	COVTESTS_(log)_		−0.070 (0.492)				0.009 (0.890)		−0.087 (0.238)	
**Containment and mitigation strategies**	DAYSWORE _(log)_	−0.136* (0.079)								
	DAYSLOCK _(log)_					0.080* (0.091)				
	INDLOCK_(log)_		0.370** (0.050)	0.256 (0.135)				0.318* (0.056)		1.066*** (0.000)
	CASBORCLO_(log)_						0.055** (0.027)			
	DAYSTEDEA				0.037 (0.648)					
	DAYDEARES								0.001** (0.029)	
**Control variables**	AGE_(log)_			0.098 (0.879)						
	GDPPC_(log)_					−0.106 (0.521)	−0.243* (0.099)			
	POV_(log)_						0.190* (0.092)			0.263** (0.017)
	RULELAW							−0.181** (0.024)		
	PERFAMREL_(log)_				0.281 (0.579)		0.300 (0.561)			
	SOCTRUST_(log)_							−0.079 (0.587)		−0.300** (0.008)
	REL_(log)_					0.191* (0.079)				
	INS					−0.342** (0.022)			− 0.327*** (0.001)	−0.451*** (0.000)
	POPDENS_(log)_							0.121* (0.057)		
	EXPPC_(log)_							0.120 (0.325)		0.105* (0.086)
	N	79	79	79	79	79	79	79	79	79
	Intercept	2.245 (0.000)	0.843 (0.233)	1.986 (0.011)	0.702 (0.464)	1.024 (0.164)	2.137 (0.037)	−1.757 (0.601)	2.039 (0.000)	0.568 (0.170)
	Pseudo *R*^2^	0.345	0.275	0.369	0.369	0.408	0.345	0.287	0.364	0.284

***Statistically significant in 1%

**Statistically significant in 5%

*Statistically significant in 10%

### Pre-pandemic healthcare conditions

The state of healthcare in place before the pandemic proved to play a significant role in developing the capacity required for the states to handle Covid-19, thereby reflecting the level of preparedness of healthcare systems. In terms of funding, health expenditure as % of GDP was found to be negatively associated with excess mortality in all the versions of the model. This finding suggests that better funded healthcare systems were less likely to be overwhelmed, responding more effectively to the pandemic and recording lower excess mortality.

Primary healthcare expenditure per capita was estimated with a negative coefficient being in line with the author’s proposition. Based on the triangulation of principles of prevention, detection and anticipation, primary healthcare could act as a catalyst in helping the system cope with the pandemic. Sufficiently funded and supported primary healthcare implies that patients with Covid-19 would be closely monitored to prevent their health deterioration, without being forced to visit hospital without a serious reason [12]. In addition, that would minimize the risk of virus contraction in healthcare workers and patients in the hospitals, who are likely to have underlying health conditions. Chiefly, a strong primary healthcare system, focusing on the care of mild cases would allow hospitals to treat both Covid-19 patients requiring hospitalization and patients suffering from other diseases [14]. In several countries, with healthcare systems getting overwhelmed, hospitals became single-disease centers, dealing only with Covid-19 patients, and, due to the government’s handling of the pandemic, being forced to neglect other patients [12]. For instance, according to NHS England, 4.9 million people were in the waiting list for hospital treatment in March 2021, up from 3.9 million in April 2020 [36]. Patients with long-term underlying health conditions were either unwilling to visit healthcare units due to concerns over getting infected or unable to attend regular or urgent medical tests as the pandemic stretched healthcare systems to their limits [12].

Health expenditure has declined during the neoliberal era [40], weakening the healthcare systems, and increasing the risk of them getting overwhelmed under special circumstances. Unsurprisingly, the per capita health expenditure % change between 2008 and 2018 proved to be adversely associated with the dependent variable. Countries that increased health expenditure per capita from 2008 to 2018, despite the 2007/08 global economic crisis, were likely to record low excess mortality, as their healthcare systems experienced improved finance conditions in the decade to 2018.

With the implementation of hollowing-out the state policies, state responsibilities have been moving sideways, expanding privatization of health care and reinforcing socio-spatial inequalities in access to health services [45]. This transition has imposed barriers against lower social classes’ access to health care, that is of great importance in the context of a pandemic [4]. The coefficient of private health expenditure as % of current health expenditure proved to be positive, suggesting that countries with extended privatization of health services could witness higher excess mortality. Apart from private health expenditure, the factor of access to healthcare was tested by employing two alternate variables without recording a change in its effect. First, the Beveridge dummy variable was found to have a negative relationship with the dependent variable. Countries using the Beveridge model were likely to experience lower excess mortality in 2020, since they tend to provide universal health coverage [51]. Second, the estimated association between the share of population covered by health insurance and excess mortality was negative as per the author’s proposition. A higher share of population covered by health insurance could improve citizens’ access to critical health services thus reducing the probability of high excess mortality.

Health expenditure, its recent trends, and the model of healthcare adopted, play a significant role in the level of healthcare resources [40]. Considering that several Covid-19 patients, particularly the most vulnerable, are expected to be hospitalized, healthcare systems with adequate resources could cope with the increased demand for specialized health services. Healthcare resources were controlled by different variables to increase the validity of results. The multivariate analysis indicated a negative association of the number of doctors, medical beds and nurses per 100 k people with excess mortality, although the relationship between the dependent variable and number of nurses, who are considered fundamental in primary care, was not statistically significant. This finding suggests better health outcomes for countries with sufficiently equipped healthcare systems.

### State capacity to handle the pandemic: the importance of test and trace

However, even relatively strong healthcare systems could be overwhelmed under emergency circumstances, without effective government strategies to handle the emergency incidents. Before explaining the analysis of these strategies, all the simulations confirm that the number of Covid-19 deaths per 100 k people was a powerful driver of excess mortality, as also showed in Fig. 3.

A fundamental difference in government responses lies in the contrast between containment and mitigation strategies. By adopting the former which is closely related to a ‘zero-Covid’ strategy, some governments acted very fast to control the pandemic and minimize its impact, recognizing the serious threat at the earliest point [39]. It was sought to eliminate the virus transmission using non-pharmaceutical interventions and achieved containment, which has been a particularly challenging endeavor in light of the large number of asymptomatic patients [50]. Alongside the imposition of border controls and clear communication strategies from the health authorities, a strong TTI capacity is considered key to such a strategy [44]. Indeed, the average index of TTI proved to be possibly the most important determinant of excess mortality across the study countries, being statistically significant and exhibiting a strong, negative association with the dependent variable. Countries demonstrating the highest average index of TTI, conducting comprehensive contact tracing for all the identified Covid-19 cases, were likely to contain the pandemic. A strong TTI capacity is of great significance in handling Covid-19, entailing that an up-to-date epidemiological system of surveillance is in place to inform policies; countries devising such a strategy, including China and New Zealand, have witnessed very low Covid-19 mortality [26, 30]. An illustrative example comes from Taiwan, a country that has coped adequately with the pandemic, recording just 1.6% excess mortality and 0.03 Covid-19 deaths per 100 k people. One of the most efficient TTI systems was implemented there, being largely backed by high tech digital tools and used to detect early transmission of Covid-19 cases and trace contacts of patients [47].

Confirming arguments suggesting that targeted testing focusing on workplaces, care homes, schools and areas of high infection risk is key to the achievement of an efficient epidemiological surveillance and strong contact-tracing capacity [12], the estimated association between the number of Covid-19 tests per 100 k people and excess mortality was not statistically significant in any version of the model. Therefore, the number of tests undertaken proved to be a less effective weapon in the battle against the pandemic, given that the only way to control the pandemic was through targeted testing in places with high infection risk [49]. By contrast, other countries, resting upon self-tests or population-wide invitations to be tested, regardless of any symptoms, had less chances to detect positive cases and, thereupon, trace their contacts, regardless of the number of tests.

Contrary to countries devising containment strategies, most governments employed mitigation policies, aspiring to prevent the number of infections exceeding the limits of the healthcare system capacity. Lockdown is considered the most restrictive strategy against the pandemic, carrying dramatic implications for employment and production, as well as dire mental health effects [3]. What might strike the reader is that the average index of stay-at-home order proved to be strongly and positively associated with excess mortality, sharply contrasting mainstream policy narratives and common beliefs. That is, countries pursuing the most stringent approach of stay-at-home orders, requiring general confinement of the population with minimal exceptions, were more likely to record high excess mortality.

In explaining the inefficiency of lockdowns to reduce excess mortality in 2020, it is suggested that countries focusing on this strategy underestimated the virus, downplayed the risks, and failed to act on time taking advantage of the time borrowed, as the first cases were officially detected at least 1 month after the outbreak of Covid-19 in China. These have not achieved to control the pandemic, since they belatedly pursued a ‘state-of-emergency’ approach to address it [54]. Having failed to contain the virus in the beginning, prioritizing the economic order, they implemented delayed horizontal lockdowns concerning the whole population in all the regions, regardless of the virus’ evolution.

Lockdown measures proved to be ineffective in that they have not been accompanied with intensive TTI and (targeted) testing. Most governments that overly relied upon lockdowns tended to neglect the importance of expanding their TTI capacity [30]. For instance, Greece went into its second lockdown on 5 November 2020, with 28 Covid-19 cases per 100 k people, but 2 weeks later these have increased to 31 [19]. Apart from the delay in taking action, this finding indicates the inertia of the Greek government to gain leverage of the time during lockdown to support the healthcare system, having chosen the strategy of stop-start lockdown, i.e. consecutive closing and opening of the economy according to the infection rate. This evidence also illustrates the lack of epidemiological surveillance in Greece, where the scientific committee set up to advise the government on the handling the pandemic recommended mitigation measures without accurate knowledge of the infection rate in the country [12]. Consequently, Greece recorded a 7.4% excess mortality in 2020 compared to the average fatalities between 2015 and 2019.

Countries that have devised a stop-start lockdown strategy, an easy solution for the government, as the main policy tool to handle Covid-19 have failed in coping well with the pandemic, while having enormous economic and social implications [1, 3]. To begin with, a late implementation of lockdown ensures that another lockdown will be applied. Indeed, most of the study countries have announced at least two periods of general confinement of the population. While valuable time was gained with the implementation of a lockdown, it was not time well spent for most governments, considering that they did not drastically reorganize the healthcare system and improve their capacity to handle the pandemic; by contrast, they resorted to partial and insufficient solutions [12]. Moreover, lockdown measures, stay-at-home orders and night curfews force the poorest people to stay in small, indoor places, thus dramatically increasing the infection risk to the rest of household members, particularly to elderly and vulnerable individuals, while restricting people to spend part of their day in open spaces where infection risk is minimized [3, 4]. Apart from the average index of stay-at-home order, the impact of lockdown strategies on excess mortality was also tested by using the total number of days of stay-at-home order. Closely related to the stop-start lockdown strategy, the duration of the general confinement of the population proved to be important. The regression analysis indicated a statistically significant and positive relationship between the total days of lockdown and excess mortality.

In light of the above, the timing of lockdown is important. The case of New Zealand provides an intriguing ground in highlighting that an early stay-at-home mandate could work on condition that the state takes advantage of the valuable time to reorganize and support effective measures to contain the virus [47]. Kapitsinis [20] has indicated that regions in countries that issued stay-at-home orders earlier have exhibited lower Covid-19 mortality rate. Germany, implementing the first lockdown 13 days after the first Covid-19 death in March 2020, was among the EU countries with the lowest Covid-19 mortality rate during the first wave of the pandemic, while also demonstrating a strong testing capacity. However, due to political pressures, the German federal government delayed the imposition of a lockdown before the outbreak of the second pandemic wave becoming widespread in October 2020, when infection rate increased from 12 per 100 k people on 21 October to 28 on 19 November 2020 [19]. Therefore, Germany recorded an excess mortality of 7.5% in 2020.

The way in which lockdowns were implemented underlines the importance of timing. The analysis supports this argument, indicating a positive relationship of excess mortality with two variables pertaining to timing: first, the number of days between the first Covid-19 death and any restriction implemented, and second, the number of Covid-19 cases per 100 k people when borders’ closure was ordered. The timing of measures’ implementation is an extremely crucial factor to their effectiveness [9]. Indeed, the efficacy of containment policies increases with their early implementation before the outbreak becomes widespread [26]. Particularly for TTI, its efficacy declines when infection rate grows quickly in a short period since contact tracing becomes infeasible [30].

Previous exposure to recent pandemics, such as SARS, MERS, avian influenza, Zika, could have incentivized government preparedness and capacity to act fast and deal with serious health events, while also improving the social acceptance and obedience to government measures, such as face masks’ use [35]. For instance, Taiwan had established an effective public health infrastructure with the National Health Command Centre to handle the SARS pandemic in 2004, which was re-activated in 2020, thus enabling early screening, efficient self-quarantine methods and massive face masks’ use, leading to the efficient containment of Covid-19 [47]. Countries with previous exposure to an epidemic were more likely to form an effective plan dealing with Covid-19.

Closely related to the timing of mitigation measures, several governments decided a premature easing of social curbs [39]. Despite the delayed response offered in the beginning, restrictions were eased before the infection rate was dealt with at a sufficient level, thereby devising the stop-start lockdown strategy. Almost all measures were lifted during summer months to accommodate the tourism industry needs paving the way to a slow-burn growth of the pandemic and leading to a sequence of outbreaks during autumn and winter months, that could had been predicted [48]. This evidence shows that there is little room for complacency about the pandemic before its end in all the countries [54].

Alongside TTI, lockdown and international travel restrictions, putting forward a Covid-19 safety agenda at the workplace proved to be crucial in this battle. The total number of days of any measure pertaining to workplace being in effect was estimated with a negative coefficient. According to the classification of the Oxford COVID-19 Government Response Tacker [15], these measures included recommendation for workplaces closure and remote working, requirement for closing businesses in specific sectors (e.g. hospitality) or order for closure of all workplaces apart from the essential firms (e.g. health services’ providers). Countries with the longest duration of workplace measures’ implementation were likely to record lower excess mortality in 2020.

### Control variables

Control variables were progressively added to the simulations to test their impact but also to enhance the robustness of the analysis of the main six factors. In terms of the level of economic growth and living conditions of the population, the analysis showed a negative relationship between GDP per capita and excess mortality, suggesting better health outcomes for countries, which are more economically developed. While wealthy areas are more globally exposed, thus increasing the likelihood of Covid-19 transmission [38], economically weak territories are associated with more acute level of deprivation and higher poverty. Indeed, poverty rate proved to be a powerful driver of excess mortality. High poverty rate could be attributed to barriers against access to healthcare, poor health conditions and limited capacity to achieve physical distancing [4]. Severe deprivation and fragile economic growth lay the explosive ground for high poverty and deep socio-economic inequalities, that are strongly related to poor health conditions [46]. Areas with high poverty could be more affected since the infection rate tends to be higher among people in lower social classes, who are forced to use public transport, in the absence of private cars, being unable to cease economic activity by virtue of low savings or work remotely due to the lack of home office or nature of their work, i.e. mainly sellers or blue-collar workers in manufacturing [4].

In terms of population features and demographics, the coefficient of population density proved to be positive. Plümper and Neumayer [42] and Biswas et al. [5] have provided similar evidence, suggesting that less densely populated areas experience a limited impact of Covid-19. The estimated relationship between median age and excess mortality was positive but not statistically significant. Countries of high median age are likely to witness high excess mortality, due to the elderly people’s susceptibility to Covid-19.

Regarding global interconnectedness, the value of exports per capita proved, as expected, to exhibit a positive coefficient, suggesting that countries with a significant position in the global production networks were likely to demonstrate high excess mortality. In line with recent findings [20], these countries are highly interconnected to other areas around the globe, having strong international trade activity, that may increase the Covid-19 mortality and thus excess deaths, due to the high mobility of people shipping commodities. This is in line with arguments that increased mobility and connectivity featured in the new globalized society accelerate the spread of a pandemic [24]. The dummy variable of insularity was estimated with a negative coefficient. Insular countries were expected to witness lower excess mortality, being better placed to control their borders and detect people infected with Covid-19 upon arrival, due to physical geography [47].

A meticulous interrogation of the formal and informal institutions offered valuable insights into the capacity of governments and society to respond effectively to the pandemic. The estimated association between the rule of law index and excess mortality was negative, suggesting better health outcomes for countries with high values of the rule of law index. The effective implementation of emergency policies could facilitate a country’s response to the pandemic and minimize (excess) mortality [7].

In terms of social norms, the interpersonal trust index was estimated with a negative coefficient, suggesting that societies with fragile trust among citizens were likely to demonstrate high excess mortality. Low stocks of social capital is likely to weaken collectivism, social solidarity and abidance by the rules, thereby escalating excess mortality [37]. This result highlights the importance of social capital, as presented by Putnam, Leonardi and Nanetti [43], who have claimed that political decisions and communication strategies are not sufficient for the success of sanitary policies, unless they are accompanied with great trust among citizens or efforts to mobilize social capital in a given community. Strong personal and family relationships may increase excess mortality, pertaining to large households, that have been indicated to escalate the Covid-19 mortality rate [20]. Nevertheless, the analysis revealed that personal and family relationships display a positive but non-significant coefficient.

Finally, the influence of religion proved to be important for excess mortality rates in 2020. The analysis highlighted that the level of weekly worship attendance is positively associated with the dependent variable. This evidence appears to be plausible, considering that religiousness observed in a country affect the epidemiological status [25]. Several religious leaders have disputed the severity of the pandemic and expressed skepticism over the very existence of Covid-19, inviting people to worship attendance. Societies with high weekly worship attendance were likely to record high excess mortality, resulting from the great concentration of people in indoor religious places, which could increase the Covid-19 infection rate.

**Fig. 4:**
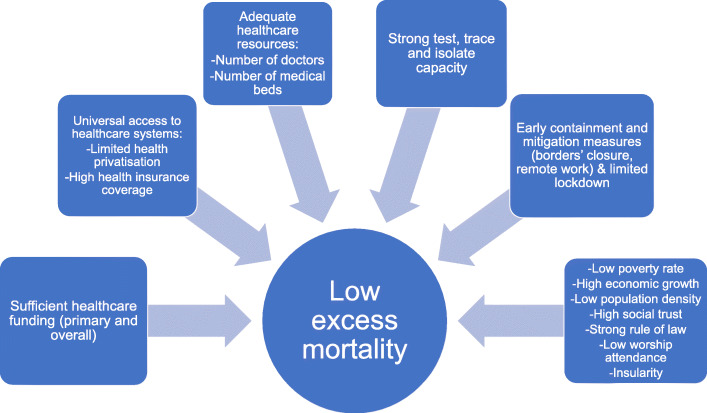
Factors that are negatively associated with excess mortality

Figure 4 summarizes the policies and aspects of the socio-economic framework that were found to be negatively associated with excess mortality. In other words, countries putting in a strong performance on these elements were more likely to exhibit low or no excess mortality in 2020, compared to the average fatalities between 2015 and 2019.

## Conclusion

This paper contributed to the emerging literature on Covid-19 effects seeking to interpret the geographical unevenness of the pandemic effects and theories the evolution of the health crisis, scrutinizing the adaptation of state capacity to manage the current pandemic. This work was one of the first to delve into the factors of excess mortality in 2020, focusing on the pre-pandemic state of healthcare and the strategies devised to handle the pandemic, across high, medium and low-income countries. The estimation of excess mortality in 2020 compared to the average fatalities observed between 2015 and 2019 indicated that, apart from the geographical unevenness in the distribution of Covid-19 deaths [23], the study countries appear to have witnessed different levels of excess fatalities. Most of them displayed excess mortality, highlighting the important impact of the pandemic. The 79 study countries recorded more than double excess deaths in 2020 compared to the officially registered Covid-19 fatalities (3.7 million to 1.5 million), underlining the significant issue of under-reporting of Covid-19 deaths.

This paper enriched our knowledge by revealing that state preparedness and capacity to handle the emergency were crucial elements for the pandemic effect in each country. The results show that poorly funded and endowed healthcare systems, having endured large-scale privatization and offering non-universal access to healthcare, alongside delayed government’s response and weak test and trace capacity, were powerful drivers of excess mortality. A brief overview of the findings comes to conclude that the level of preparedness of healthcare systems was crucial for the capacity of a country to respond effectively to the pandemic and minimize excess mortality. The research findings reveal that sufficiently funded healthcare systems, adequate level of healthcare resources (doctors, nurses, medical beds), strong primary healthcare, low rate of health privatization and wide health coverage were associated with low excess mortality in 2020. However, even relatively strong healthcare systems could be overwhelmed in the event of a serious health incident, highlighting the great significance of strategies devised by governments to handle the pandemic. The timing of application of emergency policy measures proved to be crucial, since excess mortality was positively associated with the Covid-19 infection rate following borders’ closures as well as the number of days between the imposition of any measure and first Covid-19 death. Therefore, a delayed government response, beyond a point that can prevent a spike in infections, tends to be ineffective. The results also suggest a limited benefit accrued from containment policies since the average index of stay-at-home order and the total days of lockdown were found to be positively related to excess mortality. By contrast, the duration of other strategies, such as Covid-19 safety measures at the workplace, proved to be adversely correlated with excess mortality. The average index of TTI, pertaining to a strong test, trace and isolate capacity, was found to be among the most important determinants of low excess mortality, since it improves the epidemiological surveillance, informs policies that are likely to be effective, and helps shielding the susceptible part of the population to Covid-19 infection [30].

The results about strategies devised by governments to handle the pandemic link to discourses over the feasibility and effectiveness of pursuing a zero-covid strategy as evidenced in a few, specific countries, mainly in Southeast Asia and Pacific region [39]. The findings raise the question about whether all these deaths, directly or indirectly caused by the pandemic, could have been prevented if state capacity had been greater and countries had adopted a coordinated, timely response, as claimed in a recent report of WHO [54]. The evidence presented in this paper confirms that the timing of the policy measures’ implementation was key, with countries exemplifying a timely response against uncontrolled cross-boundary movements, paving the way for efficient containment of Covid-19 [9]. The potential for containment and mitigation strategies to be effective soars if these are implemented before the outbreak becomes widespread. Beyond this point, the endeavor to control the pandemic becomes extremely challenging, with the virus bring likely to complete its cycle before becoming endemic.

However, the risk of a widespread outbreak occurring remains high even in countries that have successfully handled Covid-19, as the recent evidence from Taiwan indicates. Therefore, continuous surveillance is essential before the pandemic ends for all the countries. The poorest countries in the world, such as the ones in South America with very weak healthcare systems, inadequate staffing, insufficient health equipment and no previous experience of pandemics may exhibit the severest impact of Covid-19 before the latter turns endemic. Previous exposure to pandemics is considered to be valuable in understanding the severity of the situation and acting effectively against Covid-19 [35], with this providing positive messages for African societies.

The results indicate a relative failure of most states to cope well with the pandemic emergency, on the grounds of underestimating the severity of the virus, despite having been warned since early January 2020. Their response was far from being timely by adopting containment policies, neglecting TTI strategies and not sufficiently supporting the healthcare systems, resting all their hopes to the vaccine development. They prioritized economic order, thereby imposing and lifting late lockdowns, as well as managing rather than minimizing fatalities by devising mitigation strategies. The benefit accrued by adopting these very restrictive curbs on the spread of Covid-19 is considered insignificant [3]. There are two conditions for lockdowns to act efficient. First, it should be considered as one measure among others in the beginning of the pandemic. Second, lockdown strategies are suitable, when applied early enough to countries with low infection rates, for the support of the healthcare system. These social restrictions if applied in timely manner could assist governments to gain leverage of the lockdown period to enhance the testing capacity, facilitate remote working, prepare students’ return to schools, and genuinely support the healthcare system by reorganizing its structures, hiring additional staff, and supplying extra equipment [1]. Indeed, governments managing well with the pandemic have supported the healthcare system with additional medical staff and equipment, either directly by hiring or buying new, or indirectly, by forcing the private sector to support the public healthcare system [12].

Other elements of the socio-economic environment proved to be crucial to excess mortality in 2020. The results suggest a positive correlation of excess deaths with poverty rate and population density, while being negatively related to the level of economic growth, with high poverty and population density and weak economic growth laying the ground for severe deprivation and deep socio-economic inequalities, linking to bad health conditions and high Covid-19 infection rate [4]. Regarding formal and informal institutions, the findings reveal a negative correlation of excess mortality with social trust and the rule of law index and a positive association with worship attendance. Finally, insularity was found to be adversely associated with excess mortality.

## Data Availability

The availability of the datasets analysed during the current study is described in the following: Excess mortality in 2020 as % compared to the average mortality from 2015 to 2019 is the dependent variable. The figures about annual deaths for the years between 2015 and 2020 in 79 countries are collected from the *World Mortality Dataset* [22] and from national health authorities. Health expenditure as % of GDP in 2018 was sourced from World Bank. Primary healthcare expenditure per capita in current $ in 2018 was sourced from WHO. Per capita health expenditure % change between 2008 and 2018 is employed was sourced from World Bank. Number of doctors per 100 k people in 2018 was collected from World Bank. Number of public and private hospital beds per 100 k people in 2018 was collected from World Bank. Number of nurses and midwives per 100 k people in 2018 was collected from World Bank. Share of population covered by health insurance was sourced from Our-World-in-Data in Hasell et al. [16]. Domestic private health expenditure per capita in purchasing power parity (PPP), current international $ was sourced from WHO. Domestic private health expenditure as % of current health expenditure was sourced from WHO. Covid-19 deaths per 100 k people in 2020 were collected from Our-World-in-Data. Average index of TTI (INDTTI) for 2020 (ranging from 0 to 2) was calculated using data from the Oxford COVID-19 Government Response Tacker (OxCGRT) [15]. Covid-19 tests undertaken during 2020 per 100 k people were sourced from Our-World-in-Data. Days of any measure about workplaces (recommending or requiring closing or remote working) during 2020 were sourced from OxCGRT. Days of lockdown in 2020 were sourced from OxCGRT. Figures for the average index of stay-at-home order in 2020 were derived from OxCGRT. Covid-19 cases per 100 k people when a ban on all regions or total border closure was implemented were sourced from OxCGRT. Number of days between the detection of first Covid-19 case and first death were derived from Our-World-in-Data. Number of days between the implementation of any policy measure and first death was sourced from Our-World-in-Data. Figure for median age in 2020 were retrieved from the United Nations Database. GDP per capita, in PPP, current international $, in 2019 was retrieved from the World Bank. Poverty headcount ratio at national poverty lines in 2019 (% of population) was sourced from World Bank data. Figures for rule of law index in 2019 were retrieved from World Bank data. Personal and family relationships index in 2020 was sourced from Legatum Institute. Trust among citizens index in 2020 was sourced from Legatum Institute. Figures for the level of weekly worship attendance in 2017 were derived from Pew Research Centre. Population density figures were retrieved from United Nations data. Value of exports per capita variable in 2019 was sourced from World Bank data.

## Appendix

**Table 3:** Correlation matrix (independent variables)

	*HEAGDP*	*PRIMARPC*	*CHAHEAPC*	*DOCS*	*BEDS*	*NURS*	*HEALTHINS*	*PRIHEAPC*	*PRIHEA*	*BEVER*	*COVDEATH*	*INDTTI*	*COVTESTS*	*DAYSWORE*	*DAYSLOCK*	*INDLOCK*	*CASBORCLO*	*DAYSTEDEA*	*DAYDEARES*	*AGE*	*GDPPC*	*POV*	*RULELAW*	*PERFAMREL*	*SOCTRUST*	*REL*	*INS*	*POPDENS*	*EXPPC*
HEAGDP	1																												
PRIMARPC	0.68	1																											
CHAHEAPC	−0.17	−0.21	1																										
DOCS	0.31	0.41	−0.35	1																									
BEDS	0.06	0.23	−0.29	0.63	1																								
NURS	0.34	0.61	−0.30	0.71	0.63	1																							
HEALTHINS	0.22	0.48	−0.21	0.32	0.37	0.47	1																						
PRIHEAPC	0.57	0.82	−0.18	0.46	0.25	0.56	0.37	1																					
PRIHEAGDP	−0.16	−0.58	0.25	−0.31	− 0.24	−0.35	− 0.37	0.00	1																				
BEVER	0.28	0.43	−0.23	0.23	−0.11	0.24	0.22	0.29	−0.25	1																			
COVDEATH	0.39	0.11	−0.29	0.25	−0.08	0.05	−0.02	0.22	−0.06	− 0.06	1																		
INDTTI	−0.13	−0.04	−0.08	0.25	0.38	0.34	0.37	0.26	0.05	0.09	−0.05	1																	
COVTESTS	0.28	0.51	−0.33	0.64	0.35	0.57	0.38	0.57	−0.38	0.20	0.34	0.16	1																
DAYSWORE	0.05	0.22	0.27	0.02	0.19	−0.09	0.11	−0.11	−0.03	−0.05	−0.26	0.13	−0.14	1															
DAYSLOCK	−0.06	−0.19	0.21	−0.33	− 0.55	−0.32	− 0.30	−0.19	0.21	0.09	0.08	−0.07	−0.23	− 0.11	1														
INDLOCK	−0.20	−0.37	0.27	−0.31	− 0.42	−0.37	− 0.33	−0.21	0.37	−0.10	0.13	0.05	−0.22	−0.04	0.78	1													
CASBORCLO	0.40	0.61	−0.14	0.31	0.08	0.34	0.19	0.59	−0.19	0.15	0.22	−0.09	0.37	−0.02	−0.06	− 0.13	1												
DAYSTEDEA	0.04	0.18	−0.13	0.40	0.26	0.35	0.31	0.22	−0.18	0.34	−0.31	0.19	0.23	0.14	−0.14	−0.17	0.08	1											
DAYDEARES	0.19	0.20	0.10	−0.03	−0.03	0.11	−0.13	0.06	0.04	−0.13	−0.29	− 0.45	0.22	− 0.07	−0.07	0.18	0.36	0.17	1										
AGE	0.42	0.65	−0.37	0.67	0.69	0.65	0.46	0.56	−0.42	0.20	0.12	0.29	0.49	0.07	−0.51	−0.46	0.40	0.26	−0.02	1									
GDPPC	0.27	0.93	−0.29	0.55	0.43	0.66	0.52	0.78	−0.52	0.31	0.05	0.32	0.65	−0.14	−0.33	− 0.38	0.53	0.28	−0.08	0.69	1								
POV	0.17	−0.12	−0.14	−0.22	− 0.40	−0.34	− 0.13	−0.04	0.03	−0.06	0.37	−0.31	− 0.01	−0.11	− 0.03	−0.03	0.08	−0.11	0.02	−0.31	− 0.18	1							
RULELAW	0.47	0.90	−0.24	0.49	0.33	0.66	0.48	0.76	−0.50	0.40	−0.02	0.25	0.57	−0.13	−0.34	− 0.46	0.48	0.34	−0.04	0.66	0.89	−0.12	1						
PERFAMREL	0.21	0.35	−0.08	0.41	0.13	0.35	0.17	0.26	−0.20	0.18	0.06	−0.01	0.41	−0.12	0.05	−0.19	0.20	0.10	0.05	0.22	0.32	−0.08	0.28	1					
SOCTRUST	0.33	0.59	−0.20	0.52	0.47	0.56	0.37	0.47	−0.43	0.28	−0.13	0.14	0.41	−0.09	−0.28	− 0.38	0.35	0.37	0.06	0.56	0.61	−0.34	0.65	0.24	1				
REL	−0.08	−0.42	0.00	−0.54	−0.54	− 0.52	−0.06	− 0.24	0.25	− 0.09	0.13	− 0.19	−0.53	− 0.14	0.19	0.30	−0.16	− 0.23	0.17	− 0.55	−0.38	0.48	−0.40	− 0.28	−0.56	1			
INS	0.11	0.25	−0.08	−0.06	0.00	0.15	0.06	0.15	−0.10	0.34	−0.28	0.14	−0.07	0.00	0.06	0.01	0.02	−0.01	0.03	0.05	0.17	−0.12	0.25	0.06	0.07	0.03	1		
POPDENS	−0.03	0.11	−0.04	− 0.13	0.10	− 0.03	0.02	0.20	0.10	−0.21	−0.07	0.14	0.00	−0.08	−0.18	0.02	0.18	−0.18	−0.09	0.24	0.22	−0.09	0.16	−0.19	0.03	0.01	0.07	1	
EXPPC	0.23	0.88	−0.28	0.52	0.39	0.62	0.45	0.72	−0.52	0.26	0.05	0.25	0.74	−0.16	−0.36	− 0.43	0.54	0.25	0.03	0.67	0.95	−0.12	0.87	0.33	0.59	−0.42	0.17	0.29	1

**Table 4:** Descriptive statistics of variables used

	N	Mean	Median	ST Dev	Min	Max
EXCMOR	79	13.87	12.14	11.17	−3.97	50.42
HEAGDP	79	7.40	7.27	2.41	2.49	16.89
PRIMARPC	79	1116	694	997	34	3922
CHAHEAPC	79	35.37	28.32	49.81	−44.84	279.91
DOCS	79	286.08	289.39	127.13	35.49	635.28
BEDS	79	409.99	340	257.21	44	1305
NURS	79	726.42	670	450.45	7.37	1946.14
HEALTHINS	79	83.66	97.60	26.18	2.90	100
PRIHEAPC	79	849.01	640.90	876.88	133.75	5580.71
PRIHEA	79	36.33	34.78	14.81	12.34	72.80
BEVER	79	0.27	0	0.44	0	1
COVDEATH	79	50.41	41.80	40.80	0.03	168.90
INDTTI	79	1.34	1.43	0.43	0	1.99
COVTESTS	79	42,081	32,715	42,823	534	263,908
DAYSWORE	79	281.06	290	56.80	0	340
DAYSLOCK	79	111.49	105	92.11	0	292
INDLOCK	79	0.95	0.95	0.49	0	2.30
CASBORCLO	79	848.23	3.64	1718.58	0	7487.55
DAYSTEDEA	79	24.82	17	33.66	0	294
DAYDEARES	79	−6.46	−8	65.13	− 294	365
AGE	79	37.35	39.60	6.66	22.90	48.40
GDPPC	79	35,808	32,850	23,794	5485	124,590
POV	79	18.09	16.70	10.59	0.60	59.30
RULELAW	79	0.50	0.49	0.96	−1.18	2.02
PERFAMREL	79	77.20	79.39	9.74	37.66	91.34
SOCTRUST	79	28.04	24.80	18.26	2.50	77.40
REL	79	24.58	20	18.07	1	75
INS	79	0.14	0	0.35	0	1
POPDENS	79	330.45	93.70	1204.58	2.10	8291.90
EXPPC	79	17,082	7363	32,727	478	239,448

**Table 5:** Excess mortality (p-score) and Covid-19 deaths per 100 k people across the study countries, 2020

	**p-score**	**Covid-19 deaths per 100 k people**
**Albania**	25.72	40
**Australia**	−0.08	3.5
**Austria**	12.54	69.5
**Azerbaijan**	32.62	25.8
**Belarus**	7.97	15.1
**Belgium**	17.7	168.9
**Bolivia**	48.78	78.6
**Bosnia**	16	124.5
**Brazil**	22.36	91.9
**Bulgaria**	16.14	109.4
**Canada**	10.12	41.8
**Chile**	19.67	87.1
**China**	1.84	0.3
**Colombia**	24.44	85.4
**Costa Rica**	11.42	42.8
**Croatia**	7.95	96.4
**Cyprus**	10.31	14.4
**Czechia**	14.73	109.3
**Denmark**	2.89	22.8
**Ecuador**	49.3	79.6
**Egypt**	13.9	7.5
**El Salvador**	20.78	20.3
**Estonia**	4.05	17.6
**Finland**	2.89	10.1
**France**	10.11	95.2
**Georgia**	4.04	63.3
**Germany**	7.56	40.7
**Greece**	7.38	46.8
**Guatemala**	12.14	26.4
**Hong Kong**	6.21	1.9
**Hungary**	8.07	100
**Iceland**	3.23	8.4
**Iran**	18.91	65.8
**Ireland**	6.38	45.5
**Israel**	10.77	38.7
**Italy**	17.08	123.4
**Jamaica**	0.08	10.2
**Japan**	3.15	2.7
**Kazakhstan**	23.69	14.5
**Kyrgyzstan**	19.17	20.5
**Latvia**	1.75	34.1
**Lithuania**	10.24	67.4
**Luxembourg**	12	79
**Malaysia**	4.73	1.4
**Malta**	15.36	49.5
**Mexico**	50.42	98.1
**Moldova**	7.39	74.1
**Mongolia**	−3.97	0.03
**Netherlands**	13.73	67.8
**New Zealand**	1.15	0.5
**Nicaragua**	50.14	2.5
**Norway**	1	8
**Oman**	24.25	28.7
**Panama**	23.58	94.1
**Paraguay**	11.86	31.4
**Peru**	24.84	114.2
**Philippines**	3.74	8.4
**Poland**	20.77	76.5
**Portugal**	13.3	68.3
**Qatar**	13.79	8.5
**Romania**	15.23	82.3
**Russian Federation**	14.8	38.9
**Serbia**	12.12	47.7
**Singapore**	6.26	0.5
**Slovakia**	12.55	41.2
**Slovenia**	19.97	131.2
**South Africa**	14.06	48.7
**South Korea**	7.8	1.8
**Spain**	18.99	108.7
**Sweden**	9.61	86.4
**Switzerland**	14.08	89
**Taiwan**	1.57	0.03
**Thailand**	3.84	0.1
**Tunisia**	8.83	39.2
**Turkey**	28.2	25
**UK**	14.81	109.3
**Ukraine**	5.74	44.4
**USA**	22.74	106.9
**Uzbekistan**	12.74	1.8

## Abbreviations

WHO: Word Health Organization
TTI: Test, trace and isolate

## Footnotes

1 Excess mortality for Turkey was estimated following suggestions from Erzurumluoğlu [11].
